# Integrated multi-omics analysis reveals the molecular interplay between circadian clocks and cancer pathogenesis

**DOI:** 10.1038/s41598-023-39401-1

**Published:** 2023-08-30

**Authors:** Andy Pérez-Villa, Gabriela Echeverría-Garcés, María José Ramos-Medina, Lavanya Prathap, Mayra Martínez-López, David Ramírez-Sánchez, Jennyfer M. García-Cárdenas, Isaac Armendáriz-Castillo, Santiago Guerrero, Clara Paz, Andrés López-Cortés

**Affiliations:** 1 Cancer Research Group (CRG), Faculty of Medicine, Universidad de Las Américas, Quito, Ecuador; 2 Programa de Investigación en Salud Global, Facultad de Ciencias de la Salud, Universidad Internacional SEK, Quito, Ecuador; 3 Latin American Network for the Implementation and Validation of Clinical Pharmacogenomics Guidelines (RELIVAF-CYTED), Santiago, Chile; 4 Centro de Referencia Nacional de Genómica, Secuenciación y Bioinformática, Instituto Nacional de Investigación en Salud Pública “Leopoldo Izquieta Pérez”, Quito, Ecuador; 5 German Cancer Research Center (DKFZ), Faculty of Biosciences, Heidelberg University, Heidelberg, Germany; 6 Department of Anatomy, Saveetha Dental College and Hospital, Saveetha Institute of Medical and Technical Sciences, Chennai, India; 7 Laboratorio de Ciencia de Datos Biomédicos, Escuela de Medicina, Facultad de Ciencias Médicas de la Salud y de la Vida, Universidad Internacional del Ecuador, Quito, Ecuador; 8 Facultade de Ciencias, Universidade da Coruña, A Coruña, Spain; 9 Centro de Investigación para la Salud en América Latina (CISeAL), Pontificia Universidad Católica del Ecuador, Quito, Ecuador; 10 Grupo de Investigación Bienestar, Salud y Sociedad, Universidad de Las Américas, Quito, Ecuador

**Keywords:** Cancer genomics, Biomarkers, Data integration

## Abstract

Circadian rhythms (CRs) are fundamental biological processes that significantly impact human well-being. Disruption of these rhythms can trigger insufficient neurocognitive development, insomnia, mental disorders, cardiovascular diseases, metabolic dysfunctions, and cancer. The field of chronobiology has increased our understanding of how rhythm disturbances contribute to cancer pathogenesis, and how circadian timing influences the efficacy of cancer treatments. As the circadian clock steadily gains recognition as an emerging factor in tumorigenesis, a thorough and comprehensive multi-omics analysis of CR genes/proteins has never been performed. To shed light on this, we performed, for the first time, an integrated data analysis encompassing genomic/transcriptomic alterations across 32 cancer types (n = 10,918 tumors) taken from the PanCancer Atlas, unfavorable prognostic protein analysis, protein–protein interactomics, and shortest distance score pathways to cancer hallmark phenotypes. This data mining strategy allowed us to unravel 31 essential CR-related proteins involved in the signaling crossroad between circadian rhythms and cancer. In the context of drugging the clock, we identified pharmacogenomic clinical annotations and drugs currently in late phase clinical trials that could be considered as potential cancer therapeutic strategies. These findings highlight the diverse roles of CR-related genes/proteins in the realm of cancer research and therapy.

## Introduction

**Figure 1:**
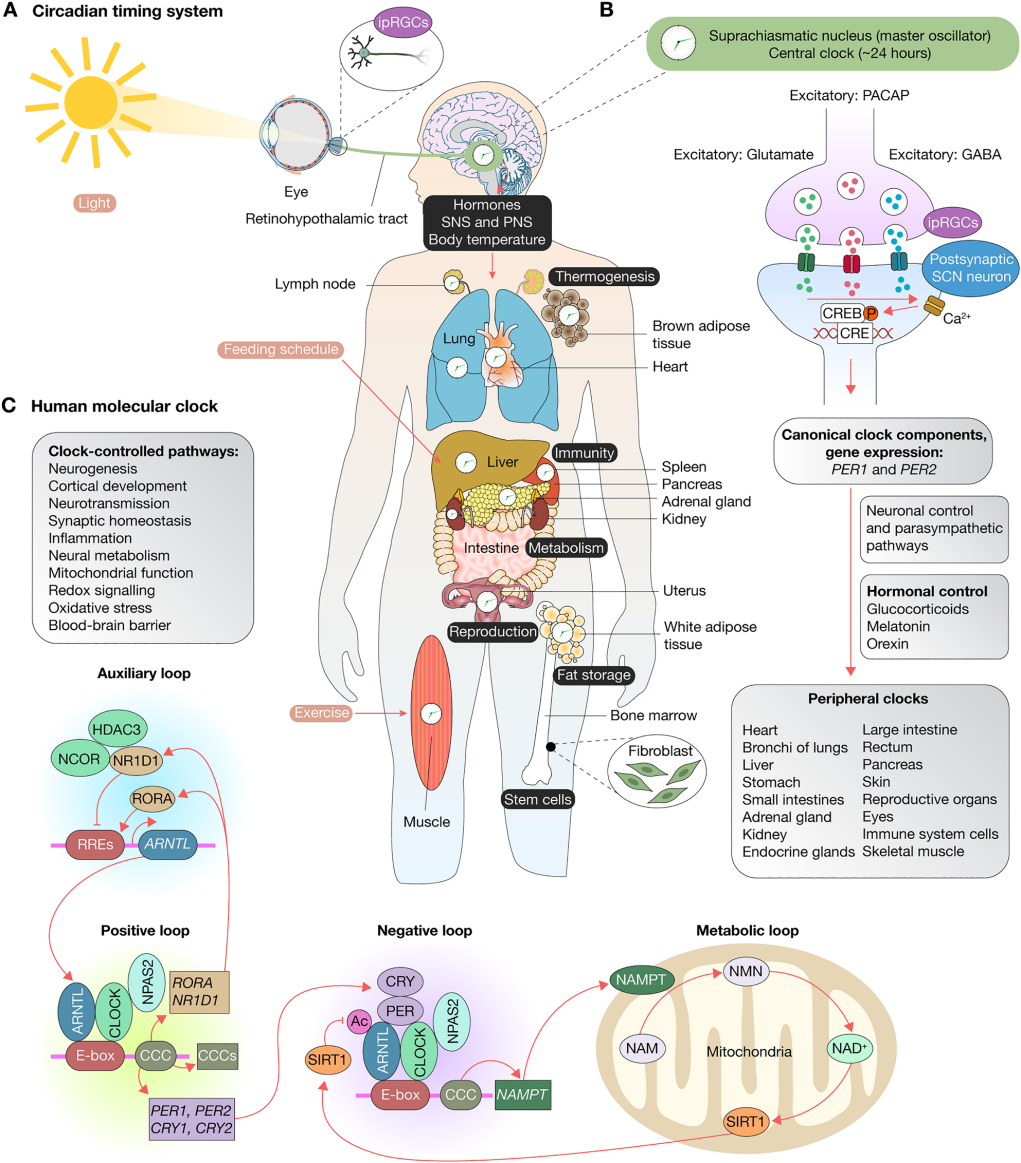
The circadian rhythms and circadian clock. (**A**) The circadian timing system synchronizes central and peripheral clocks across the human body to adapt our physiology to environmental changes. Light is received by ipRGCs in the eyes, which sends electrical signals to the SCN through the retinohypothalamic tract. The peripheral nervous system and humoral signals convey information from the SCN to orchestrate peripheral clocks. Feeding schedule and exercise can also activate central and peripheral clocks. Finally, circadian rhythms regulate hormones, thermogenesis, immunity, metabolism, reproduction, fat storage, and stem cell development. (**B**) Neurotransmitters released by ipRGCs. Glutamate and PACAP cause membrane depolarization in the postsynaptic SCN neurons. Changes in cAMP and Ca^2+^ levels induce phosphorylation of the CREB protein, and expression of canonical clock components (i.e., *PER1* and *PER2*), thereby resetting SCN cellular oscillators. GABA, an inhibitory neurotransmitter, decreases the sensitivity of non-image-forming behaviors at low light levels. Lastly, SCN neurons control peripheral clocks throughout the body via neuronal and hormonal signals. (**C**) The human molecular clock is composed of canonical clock components, clock-controlled genes, and clock-controlled pathways. The clock is operated through a network of transcription-translation feedback loops (positive, negative, auxiliary, and metabolic) that oscillate with a near-24-h cycle (see Introduction). *ipRGC* intrinsically photosensitive retinal ganglion cells, *SCN* suprachiasmatic nucleus, *PACAP* pituitary adenylate cyclase-activating polypeptide, *GABA* γ-aminobutyric acid, *RRE* RORA or NR1D1 response elements, *CCC* canonical clock components, *NAM* nicotinamide, *NMN* nicotinamide mononucleotide, *NAD*^*+*^ nicotinamide adenine dinucleotide.

Circadian rhythms (CRs) are near-24-h oscillations that regulate a broad range of physiological and behavioral processes, including sleep–wake cycles, cell cycle gating, mitochondrial function, DNA damage repair, cellular redox, hypoxia, autophagy, apoptosis, and immune function^1–3^. These rhythms are guided by central and peripheral biological clocks, which are influenced by external cues known as zeitgebers (German for “time givers''), such as light, temperature, and feeding schedule^4^. Light signals, in particular, are received by specialized melanopsin-producing photoreceptive retinal ganglion cells (ipRGCs) in the eyes and conveyed to the central pacemaker, the suprachiasmatic nucleus (SCN), in the anterior hypothalamus^5^. The SCN relays timing information to various brain regions, enabling the orchestration of peripheral clocks located in nearly all body cells^6–8^ (Fig. 1A). While these peripheral clocks can be influenced by the SCN through neurotransmitters, endocrine factors, and bodily fluids^9^ (Fig. 1B), they can also function independently, adjusting to non-light zeitgebers. Consequently, there is a feedback mechanism between SCN and peripheral clocks, ensuring synchronized CRs across the body^10^.

Circadian rhythms consist of three major groups of genes/proteins: (a) the canonical clock components (CCCs), which directly and crucially contribute to the generation and maintenance of circadian rhythms^11^; (b) the clock-controlled genes/proteins (CCGs/CCPs), which act as regulatory nodes in specific cell types and have their expression influenced by the circadian clock^12^; and (c) the genes/proteins involved in mediating the neural mechanisms of circadian rhythmicity and its entrainment (NMCRE), which function as nodes in neural and physiological processes that regulate CRs and synchronize them with external cues^13^.

The human molecular clock is comprised of CCCs, including *ARNTL*, *ARNTL2*, *CLOCK*, *CRY1*, *CRY2*, *CSNK1D*, *CSNK1E*, *NPAS2*, *NR1D1*, *NR1D2*, *PER1*, *PER2*, *PER3*, *RORA*, *RORB*, *RORC*, and *TIMELESS*^10^. This molecular clock operates at a single-cell level through transcription-translation feedback loops that oscillate on a near-24-h cycle. The positive loop is initiated by the interaction of CLOCK or NPAS2 with ARNTL in the nucleus. The resulting heterodimers bind to E-boxes located in the promoter regions of CCCs to regulate their transcription^14^. As PER and CRY proteins reach a certain cytoplasmic concentration, they are transported to the nucleus to interact with the ARNTL/CLOCK complex, inhibiting their own transcription, thereby creating a negative feedback loop^14^. An auxiliary loop is involved in the transcription of *ARNTL* by activating or inhibiting the RORA or NR1D1 response elements (RREs), respectively^2^. Additionally, the ARNTL/CLOCK complex controls the expression of NAMPT, a key enzyme in the mitochondrial metabolic loop to inhibit the CLOCK-driven transcription (Fig. 1C).

Disturbances in CRs, induced by factors such as widespread use of electric light at night, sleep deprivation, night shift work, chronic jet lag, or nocturnal eating habits, are closely associated with impaired neurocognitive development, sleep and mood disorders, insomnia, mental disorders, cardiovascular diseases, metabolic diseases, and cancer^5,15–18^. Particularly, alterations in CRs due to genetic, environmental or pathological risk factors can significantly alter the expression and function of tumor suppressors, oncogenes, DNA repair, apoptotic, and immune-related genes in both host and tumor tissues, potentially favoring the onset and progression of cancer^3,19^. Such chrono-disruptions can lead to uncontrolled proliferation, metastatic spread, inflammation, escaping apoptosis, immune evasion, enhanced angiogenesis, and anti-cancer drug resistance^3^. Therefore, maintaining a well-regulated circadian rhythm could be a crucial strategy in cancer prevention^20,21^.

Recently, Sulli et al*.* proposed three distinct categories for chronotherapeutic approaches: (a) training the clock to enhance a robust circadian rhythm in feeding-fasting, sleep–wake, or light–dark cycles; (b) clocking the drugs, which involves optimizing the timing of drug administration to maximize efficacy and minimize adverse side effects; and (c) drugging the clock, which employs agents targeting the circadian clock^5^. Nonetheless, the discovery of therapeutic targets and effective drugs involved in the CRs of cancer remains limited. To address this gap, we performed, for the first time, an integrated multi-omics data analysis, encompassing the assessment of genomic and transcriptomic alterations of CR-related genes across 32 cancer types sourced from the PanCancer Atlas (PCA) project of The Cancer Genome Atlas (TCGA) consortium^22^. We also identified CR-related genes associated with unfavorable prognosis based on the Human Pathology Atlas^23^, designed a protein–protein interactome network, and defined the shortest pathways to cancer hallmark phenotypes. Collectively, these approaches led to the discovery of potential therapeutic targets, pharmacogenomic clinical annotations, and drugs currently in late stage clinical trials. These findings should be considered to enhance the effectiveness of cancer chronotherapy.

## Results

### OncoPrint of genomic and transcriptomic alterations according to the TCGA PanCancer Atlas

**Figure 2:**
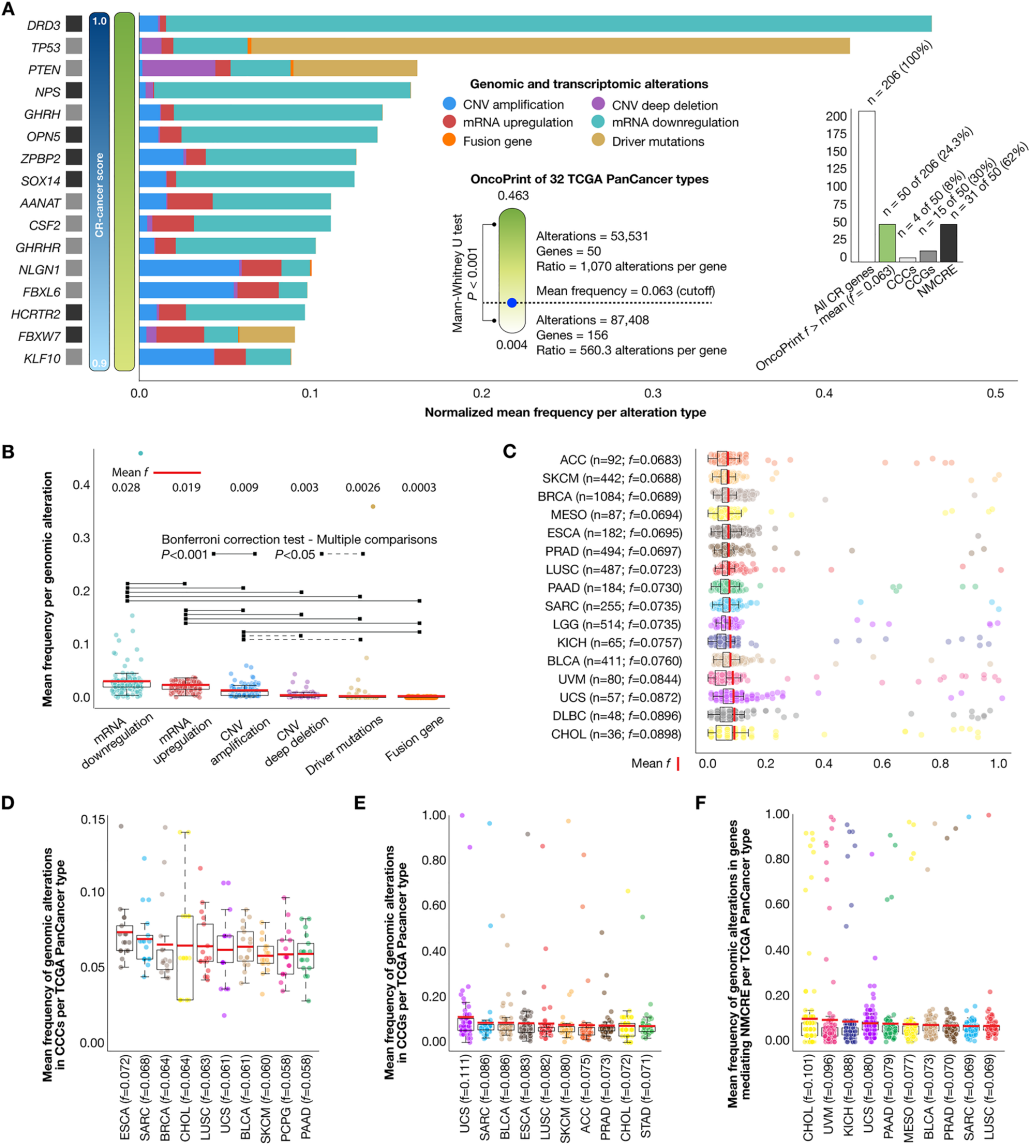
OncoPrint of genomic and transcriptomic alterations across 32 TCGA PanCancer types. (**A**) Ranking of the most altered CR-related genes (n = 206) considering the mean *f* of alteration events (cutoff = 0.063) and the CR-cancer score > 0.9. The Mann–Whitney U test showed significant difference of genomic alterations between genes upper the mean *f* and lower the mean *f* (*P* < 0.001). The OncoPrint was performed using the data from the cBioPortal platform (http://www.cbioportal.org/)^63,64^. (**B**) Mean *f* per alteration type and significant Bonferroni correction (*P* < 0.001) of mRNA downregulation, mRNA upregulation and CNV amplification in comparison with other alterations. (**C**) Ranking of the 50% (n = 16) most altered TCGA PanCancer types according to the mean *f* of alterations. (**D**) Ranking of the top ten TCGA PanCancer types with highest mean *f* of genomic alterations in CCCs. (**E**) Ranking of the top ten TCGA PanCancer types with highest mean *f* of genomic alterations in CCGs. (**F**) Ranking of the top ten TCGA PanCancer types with highest mean *f* of genomic alterations in genes mediating NMCRE. *CR* circadian rhythm, *CCCs* canonical clock components, *CCGs* clock-controlled genes, *NMCRE* neural mechanisms of circadian rhythmicity and its entrainment, *CNV* copy number variants, *TCGA* The Cancer Genome Atlas, *mRNA* messenger RNA, *BRCA* breast invasive carcinoma, *LGG* brain lower grade glioma, *PRAD* prostate adenocarcinoma, *LUSC* lung squamous cell carcinoma, *SKCM* skin cutaneous melanoma, *STAD* stomach adenocarcinoma, *BLCA* bladder urothelial carcinoma, *SARC* sarcoma, *PAAD* pancreatic adenocarcinoma, *ESCA* esophageal carcinoma, *ACC* adrenocortical carcinoma, *MESO* mesothelioma, *UVM* uveal melanoma, *KICH* kidney chromophobe, *UCS* uterine carcinosarcoma, *DLBC* lymphoid neoplasm diffuse large B-cell lymphoma, *CHOL* cholangiocarcinoma.

After obtaining sets of CCCs (n = 17), CCGs (n = 59), genes involved in mediating the NMCRE (n = 130), and cancer driver genes (n = 873) (Supplementary Tables 1 and 2), we identified 140,939 genomic and transcriptomic alteration events in the entire set of CR-related genes (n = 206) belonging to 10,918 individuals across 32 different TCGA PanCancer types. Figure 2A and Supplementary Tables 3 to 35 detailed the OncoPrint of alterations, including mRNA upregulation, mRNA downregulation, copy number variant (CNV) deep deletion, CNV amplification, fusion gene, and driver mutations, involving 1084 (9.9%) individuals with breast invasive carcinoma (BRCA), 594 (5.4%) with colorectal carcinoma (CRC), 585 (5.4%) with ovarian serous cystadenocarcinoma (OV), 585 (5.4%) with glioblastoma multiforme (GBM), 566 (5.2%) with lung adenocarcinoma (LUAD), 529 (4.8%) with uterine corpus endometrial carcinoma (UCEC), 523 (4.8%) with head and neck squamous cell carcinoma (HNSC), 514 (4.7%) with brain lower grade glioma (LGG), 512 (4.7%) with kidney renal clear cell carcinoma (KIRC), 499 (4.6%) with thyroid carcinoma (THCA), 494 (4.5%) with prostate adenocarcinoma (PRAD), 487 (4.5%) with lung squamous cell carcinoma (LUSC), 442 (4.0%) with skin cutaneous melanoma (SKCM), 440 (4.0%) with stomach adenocarcinoma (STAD), 411 (3.8%) with bladder urothelial carcinoma (BLCA), 372 (3.4%) with liver hepatocellular carcinoma (LIHC), 297 (2.7%) with cervical squamous cell carcinoma and endocervical adenocarcinoma (CESC), 283 (2.6%) with kidney renal papillary cell carcinoma (KIRP), 255 (2.3%) with sarcoma (SARC), 184 (1.7%) with pancreatic adenocarcinoma (PAAD), 182 (1.7%) with esophageal carcinoma (ESCA), 178 (1.6%) with pheochromocytoma and paraganglioma (PCPG), 165 (1.5%) with acute myeloid leukemia (LAML), 149 (1.4%) with testicular germ cell tumors (TGCT), 123 (1.1%) with thymoma (THYM), 92 (0.8%) with adrenocortical carcinoma (ACC), 87 (0.8%) with mesothelioma (MESO), 80 (0.7%) with uveal melanoma (UVM), 65 (0.6%) with kidney chromophobe (KICH), 57 (0.55) with uterine carcinosarcoma (UCS), 48 (0.4%) with lymphoid neoplasm diffuse large B-cell lymphoma (DLBC), and 36 (0.3%) with cholangiocarcinoma (CHOL).

After normalizing the frequency (*f*) of alteration events, which involved dividing the number of alteration events per gene by the number of individuals in each cancer cohort, the overall analysis revealed that 50 (24.3%) CR-related genes were significantly altered (Mann–Whitney U test *P* < 0.001) with alteration *f* higher than the average (cutoff > 0.063). Among them, 4 (8%) were CCCs, 15 (30%) were CCGs, and 31(62%) were genes involved in mediating the NMCRE. The CR-related genes with both the highest *f* of alteration events and the highest CR-cancer scores (cutoff > 0.9) were *DRD3*, *TP53*, *PTEN*, *NPS*, *GHRH*, *OPN5*, *ZPBP2*, *SOX14*, *AANAT*, *CSF2*, *GHRHR*, *NLGN1*, *FBXL6*, *HCRTR2*, *FBXW7*, and *KLF10*.

The most common alteration type, with a mean *f* of 0.028, was mRNA downregulation, followed by mRNA upregulation (*f* = 0.019), CNV amplification (*f* = 0.009), CNV deep deletion (*f* = 0.003), putative driver mutations (*f* = 0.0026), and fusion genes (*f* = 0.0003). We performed the Bonferroni correction as a multiple comparison test to obtain significant alterations (*P* < 0.001) across the TCGA PanCancer types. We found that mRNA downregulation, mRNA upregulation, and CNV amplification were significantly altered (*P* < 0.001) across genomic and transcriptomic alterations (Fig. 2B).

Figure 2C shows the TCGA PanCancer types with the highest mean alteration frequencies into CR-related genes. The top three cancer types with the highest mean *f* of genomic and transcriptomic alteration events were CHOL (*f* = 0.089), DLBC (*f* = 0.089), and UCS (*f* = 0.087) (Supplementary Table 36). Moreover, the top three TCGA PanCancer types with the highest *f* of genomic alterations in CCCs were ESCA (*f* = 0.072), SARC (*f* = 0.068), and BRCA (*f* = 0.064) (Fig. 2D); in CCGs were UCS (*f* = 0.111), SARC (*f* = 0.086), and BLCA (*f* = 0.086) (Fig. 2E); and, in genes mediating NMCRE were CHOL (*f* = 0.101), UVM (*f* = 0.096), and KICH (*f* = 0.088) (Fig. 2F).

### Circadian rhythm protein–protein interactome network

**Figure 3:**
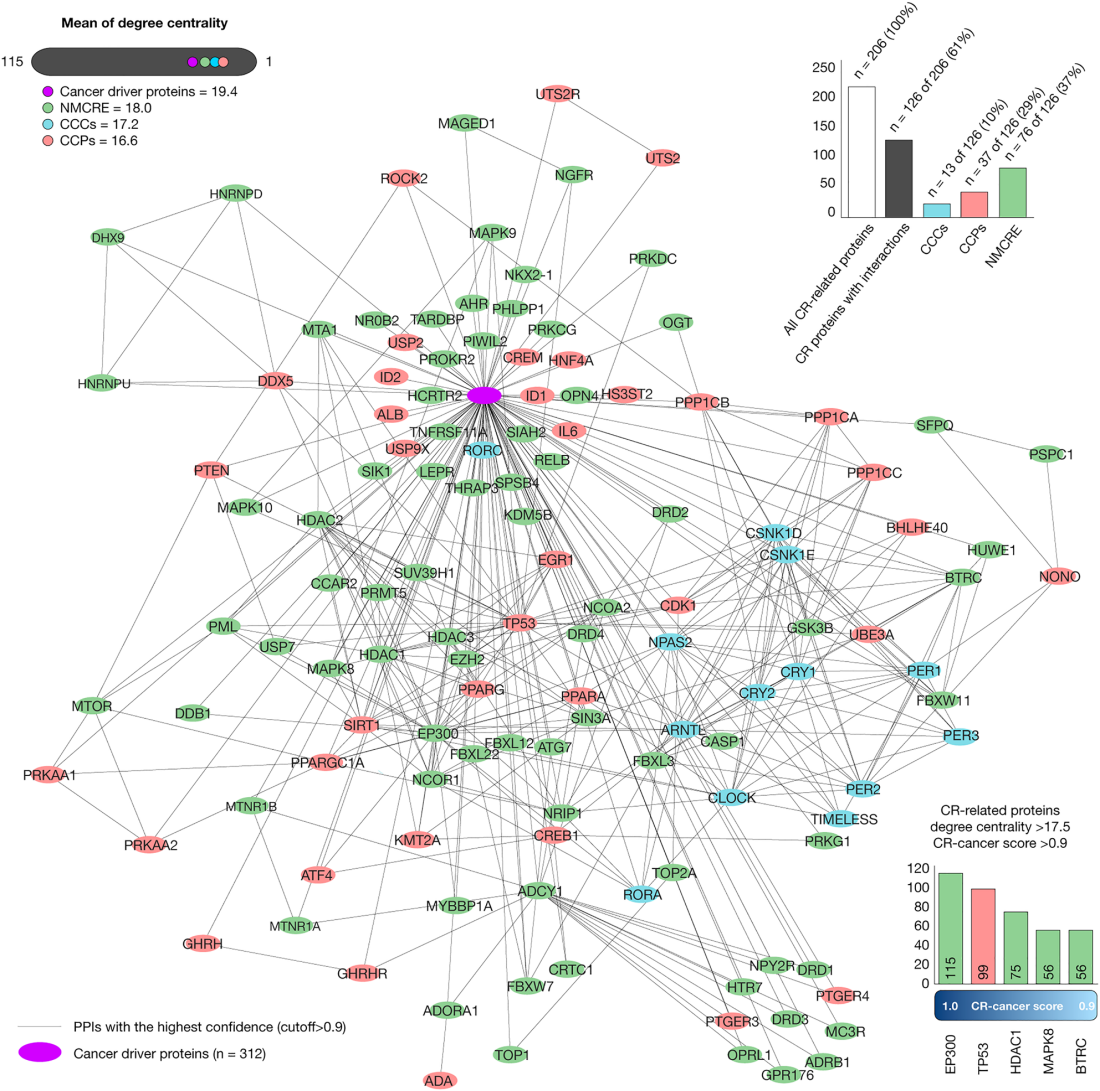
Circadian rhythm protein–protein interactome network. Network made up of 13 CCCs (sky blue nodes; mean degree centrality = 17.2), 37 (29%) CCPs (red nodes; mean degree centrality = 16.6), and 76 (37%) proteins involved in the NMCRE (green nodes; mean degree centrality = 18.0) with at least one high-confidence interaction (cutoff > 0.9) with cancer driver proteins (pink node; mean degree centrality = 19.4). The Mann–Whitney U test showed a correlation of degree centrality between cancer driver nodes and CR-related nodes (*P* > 0.05). The CR-related proteins with both the highest degree centrality and the CR-cancer scores > 0.9 were EP300, TP53, HDAC1, MAPK8, and BTRC. Lastly, the CR-PPi network was designed and visualized through the Cytoscape software v.3.10 (https://cytoscape.org/)^72^. *CR* circadian rhythm, *PPi* protein–protein interaction, *CCCs* canonical clock components, *CCPs* clock-controlled proteins, *NMCRE* neural mechanisms of circadian rhythmicity and its entrainment.

The CR protein–protein interactome network (CR-PPi) was generated to better understand the connectivity between CRs and cancer through high-confidence interactions (cutoff > 0.9). Figure 3 shows the CR-PPi network encompassed by 412 (100%) nodes and 3795 high-confidence edges. Of these nodes, 312 (76%) were cancer driver proteins with a mean of degree centrality of 19.4, 13 (10%) were CCCs with a mean of 17.2, 37 (29%) were CCPs with a mean of 16.6, and 76 (37%) were proteins involved in mediating the NMCRE with a mean of 18.0. Lastly, the CR-related proteins with both the highest degree centrality and the highest CR-cancer scores (cutoff > 0.9) were EP300, TP53, HDAC1, MAPK8, and BTRC (Supplementary Table 37).

### A pathology atlas of the human cancer transcriptome

**Figure 4:**
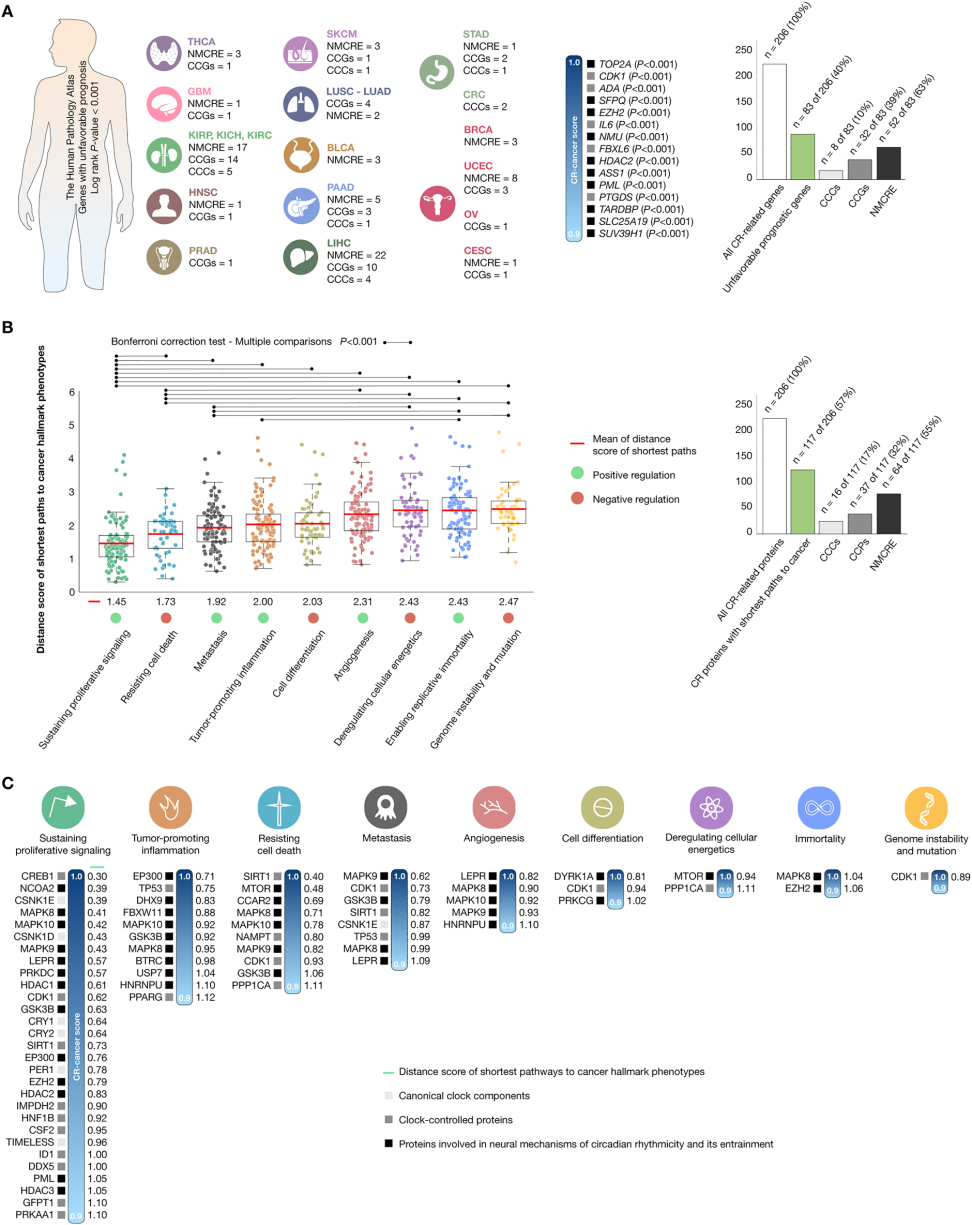
Circadian rhythm-related genes/proteins with unfavorable prognosis in different cancer types and distance score of shortest pathways to cancer hallmark phenotypes. (**A**) The Human Pathology Atlas details the CR-related genes with unfavorable prognosis and log rank *P*-value < 0.001 across 19 cancer types. Additionally, 15 CR-related genes had the highest CR-cancer scores (> 0.9). All data was taken from The Human Protein Atlas platform (https://www.proteinatlas.org/)^23^. (**B**) Box plots showing the mean distance scores of shortest paths per cancer hallmark phenotype, and the Bonferroni correction as multiple comparison test (*P* < 0.001) to show significant differences across cancer phenotypes. The shortest paths to cancer hallmark phenotype analysis reveals that 16 (14%) CCCs, 37 (32%) CCPs, and 64 (55%) proteins involved in the NMCRE have the shortest distance scores to cancer phenotypes. (**C**) CR-related proteins with both the shortest distance scores to cancer hallmark phenotypes and the highest CR-cancer scores (> 0.9). Lastly, the shortest paths to cancer hallmark phenotypes were analyzed by using data from CancerGeneNet (https://signor.uniroma2.it/CancerGeneNet/)^24^. *CR* circadian rhythm, *CCCs* canonical clock components, *CCPs* clock-controlled proteins, *NMCRE* neural mechanisms of circadian rhythmicity and its entrainment, *THCA* thyroid carcinoma, *GBM* glioblastoma multiforme, *HNSC* head and neck squamous cell carcinoma, *LUSC* lung squamous cell carcinoma, *LUAD* lung adenocarcinoma, *BRCA* breast invasive carcinoma, *UCEC* uterine corpus endometrial carcinoma, *OV* ovarian serous cystadenocarcinoma, *CESC* cervical squamous cell carcinoma and endocervical adenocarcinoma, *KIRP* kidney renal papillary cell carcinoma, *KICH* kidney chromophobe, *KIRC* kidney renal clear cell carcinoma, *LIHC* liver hepatocellular carcinoma, *PAAD* pancreatic adenocarcinoma, *SKCM* skin cutaneous melanoma, *PRAD* prostate adenocarcinoma, *BLCA* bladder urothelial carcinoma, *STAD* stomach adenocarcinoma, *CRC* colorectal carcinoma.

After exploring the Human Pathology Atlas created by the Human Protein Atlas program, we conducted a Kaplan–Meier analysis to examine the correlation between mRNA expression levels of CCCs, CCGs, and genes involved in mediating the NMCRE with patient survival. This analysis aimed to determine the prognostic significance of each CR protein-coding gene across 17 TCGA PanCancer types, encompassing nearly 8000 patients. The findings highlight the efficacy of large-scale system biology endeavors that leverage publicly available resources. In this context, we identified 83 CR-related genes that exhibited unfavorable prognostic significance (significant log rank *P*-value < 0.001) in 16 TCGA cancer types. Among these genes, 8 (10%) were CCCs, 32 (39%) were CCGs, and 52 (63%) were involved in the NMCRE. Lastly, the most significant CR-related genes with a CR-cancer score > 0.9 were *TOP2A*, *CDK1*, *ADA*, *SFPQ*, *EZH2*, *IL6*, *NMU*, *FBXL6*, *HDAC2*, *ASS1*, *PML*, *PTGDS*, *TARDBP*, *SLC25A19*, and *SUV39H1* (Fig. 4A and Supplementary Table 38).

### Shortest pathways from circadian rhythm proteins to cancer hallmark phenotypes

We utilized CancerGeneNet software to analyze a set of 206 CR-related proteins and observed that 117 (57%) of them exhibited distance scores indicating their involvement in the shortest pathways to cancer hallmark phenotypes^24^. Among these proteins, 16 (14%) were CCCs, 37 (32%) were CCPs, and 64 (55%) were proteins involved in mediating NMCRE. In Fig. 4B, we presented box plots illustrating the CR-related proteins with shortest paths to various cancer hallmark phenotypes, with the top three being cell proliferation (mean distance score = 1.45; number of proteins = 104), resisting cell death (1.73; 52), and metastasis (1.92; 107). Through the Bonferroni correction test, we observed that CR-related proteins had significantly shorter paths (*P* < 0.001) among these cancer hallmark phenotypes. Additionally, Fig. 4C highlights the 42 CR-related proteins with both the shortest paths to cancer hallmark phenotypes and the highest CR-cancer scores (cutoff > 0.9). Among them, CREB1 was highly connected to cell proliferation, EP300 to inflammation, SIRT1 to resisting cell death, MAPK9 to metastasis, LEPR to angiogenesis, DYRK1A to cell differentiation, MTOR to deregulating cellular energetics, MAPK8 to immortality, and CDK1 to genome instability (Supplementary Table 39).

### Multi-omics data integration and functional enrichment analysis

**Figure 5:**
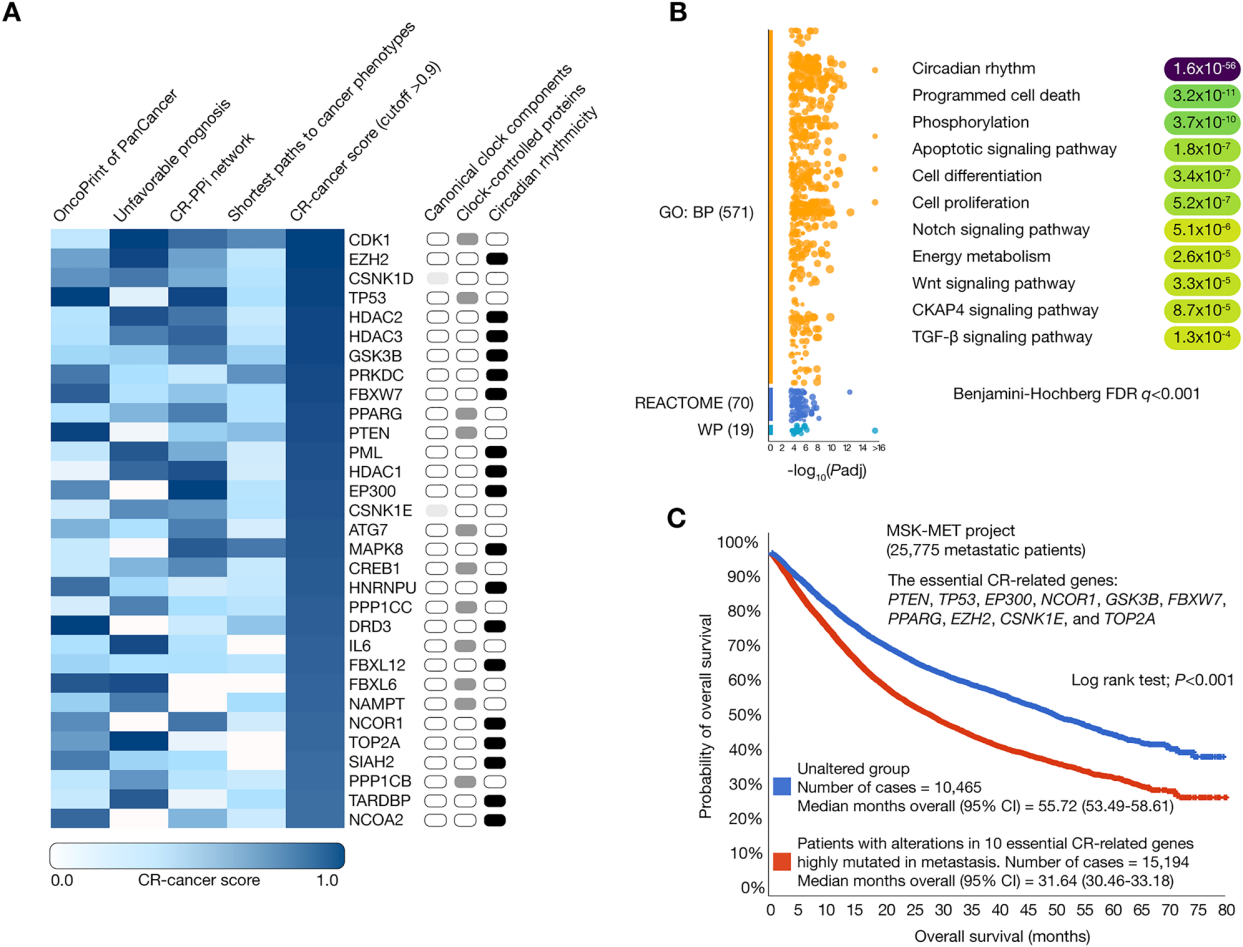
Integrated multi-omics data analysis and functional enrichment analysis. (**A**) Heatmap of CR-cancer scores per multi-omics approach to prioritize the 31 essential CR-related proteins significantly involved in cancer (cutoff > 0.9). (**B**) The functional enrichment analysis displays the most significant annotations (Benjamini–Hochberg *FDR* < 0.01) related to the GO biological processes (http://geneontology.org/)^55^, the Reactome signaling pathways (https://reactome.org/)^83^, and the WikiPathways (https://www.wikipathways.org/)^84^ on cancer. The results of this enrichment were visualized using a Manhattan plot and were obtained through the g:Profiler software version e101_eg48_p14_baf17f0 (https://biit.cs.ut.ee/gprofiler/gost)^25^. (**C**) Overall survival analysis comparing metastatic patients with genomic alterations in 10 essential CR-related genes (n = 15,194) versus unaltered patients (n = 10,465). Unaltered patients had a median month average (55.72) significantly higher than altered patients (31.64) showing a log rank test *P* < 0.001. *CR* circadian rhythm, *GO* gene ontology, *WP* WikiPathways, *BP* biological processes, *FDR* false discovery rate, *MSK-MET* Memorial Sloan Kettering–Metastatic Events and Tropism, *CI* confidence intervals.

Figure 5A illustrates a heatmap of CR-cancer scores per multi-omics approach to prioritize the 31 essential CR-related proteins significantly involved in cancer (cutoff > 0.9) (Supplementary Table 40). Among them, 2 (7%) were canonical clock components (CSNK1D and CSNK1E), 11 (35%) were clock-controlled proteins (CDK1, TP53, PPARG, PTEN, ATG7, CREB1, PPP1CC, IL6, FBXL6, NAMPT, and PPP1CB), and 18 (58%) were proteins involved in mediating neural mechanisms of circadian rhythmicity and its entrainment (EZH2, HDAC2, HDAC3, GSK3B, PRKDC, FBXW7, PML, HDAC1, EP300, MAPK8, HNRNPU, DRD3, FBXL12, NCOR1, TOP2A, SIAH2, TARDBP, and NCOA2).

Subsequently, we conducted a functional enrichment analysis of the 31 essential CR-related proteins significantly involved in cancer by using the g:Profiler software^25^. The Manhattan plot helped us to identify 571 gene ontology (GO) biological processes, 70 Reactome signaling pathways, and 19 WikiPathways. Interestingly, this enrichment analysis displayed a strong correlation with several cancer hallmark phenotypes and cancer signaling pathways. The most significant biological annotations, with Benjamini–Hochberg correction and false discovery rate (FDR) *q* < 0.001, included programmed cell death, phosphorylation, apoptotic signaling pathway, cell differentiation, cell proliferation, Notch signaling pathway, energy metabolism, Wnt signaling pathway, CKAP4 signaling pathway, and TGF-β signaling pathway (Fig. 5B and Supplementary Table 41).

### Identification of essential circadian rhythm-related genes highly altered in a metastatic cohort

Memorial Sloan Kettering–Metastatic Events and Tropism (MSK–MET) represents an integrated PanCancer cohort comprising tumor genomic and clinical outcome data from 25,775 patients, highlighting patterns of metastatic dissemination across 50 tumor types^26^. Interestingly, 10 of our 31 essential CR-related genes (*PTEN*, *TP53*, *EP300*, *NCOR1*, *GSK3B*, *FBXW7*, *PPARG*, *EZH2*, *CSNK1E* and *TOP2A*) exhibited genomic alterations in the metastatic cohort^27^. Subsequently, we carried out an overall survival analysis contrasting patients with genomic alterations in these 10 essential CR-related genes (n = 15,194) against those without alterations (n = 10,465). Consequently, the group with alterations demonstrated a median overall survival (95% coefficient intervals) of 31.64 (30.46–33.18) months, while the group without alterations had a median overall survival of 55.72 (53.49–58.61) months. The log rank test revealed a statistically significant difference (*P* < 0.001) in overall survival months when comparing altered and unaltered patients (Fig. 5C).

### Pharmacogenomic clinical annotations of circadian rhythm genes in cancer

**Figure 6:**
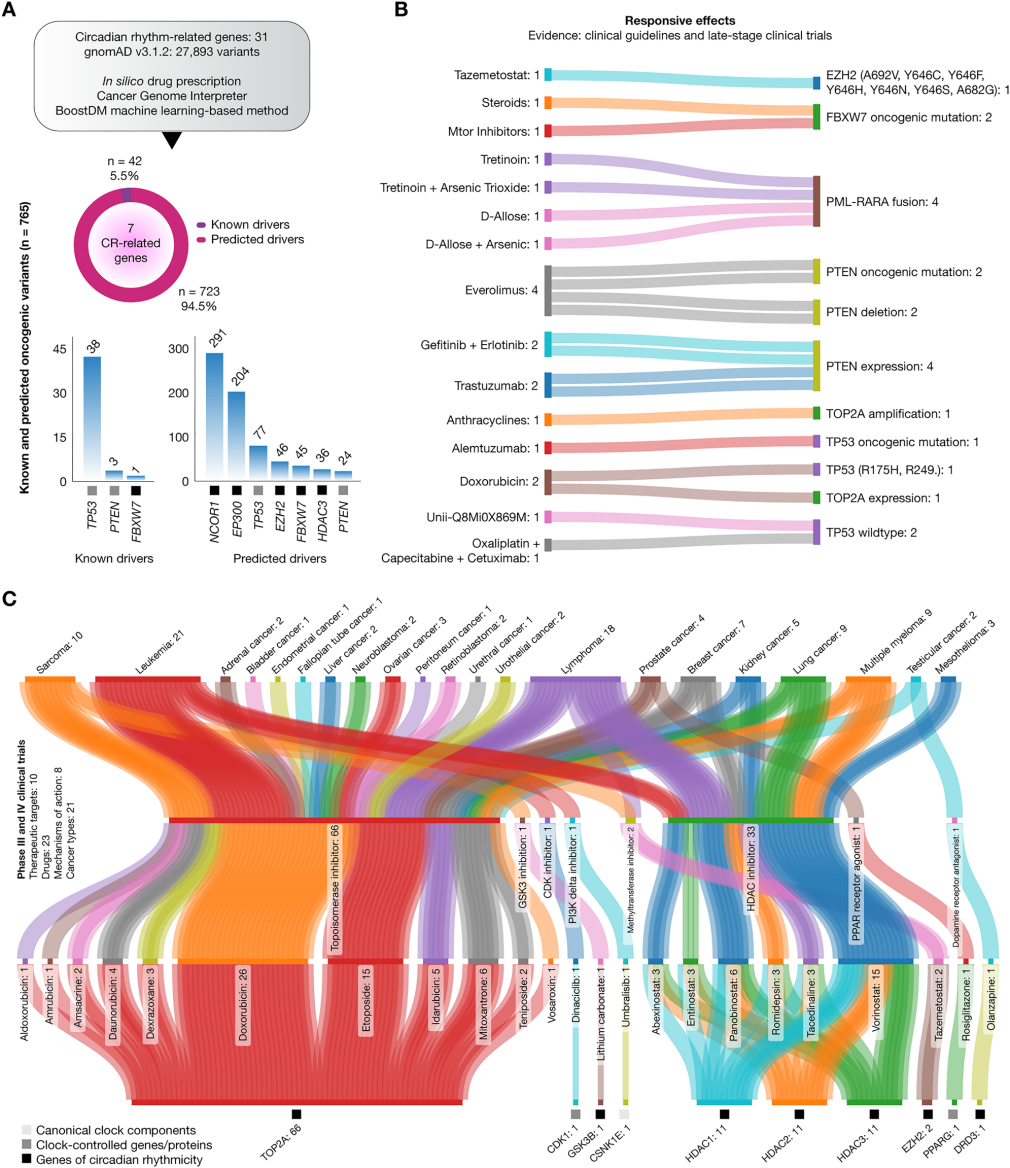
Pharmacogenomic clinical annotations and clinical trials. (**A**) Identification of known and predicted oncogenic variants (n = 765) through the boostDM machine learning-based method^49^. (**B**) Sankey plot of in silico drug prescription based on pharmacogenomic clinical annotations. **(C)** Overview of phase III and IV clinical trials. Sankey plot showing therapeutic targets, drugs, mechanisms of action, and cancer types involved in late phase clinical trials. Data of clinical trials and mechanisms of action were taken from the Open Targets Platform (https://platform.opentargets.org/)^29^, and the Drug Repurposing Hub (https://clue.io/repurposing)^89^. Lastly, Sankey plots were designed using the SankeyMATIC software (https://sankeymatic.com/ and https://github.com/nowthis/sankeymatic) *CR* circadian rhythm.

Figure 6A,B display the panoramic landscape of cancer pharmacogenomic strategies where CR-related genes were involved. Figure 6A presents the results of the boostDM analysis aimed to identify oncogenic variants into the 31 essential CR-related genes strongly associated with cancer. Following the analysis of 27,893 single nucleotide variants and insertion/deletion variants, we pinpointed 765 oncogenic variants (Supplementary Table 42). Among these, 42 (5.5%) were known drivers carried by *TP53* (91%), *PTEN* (7%), and *FBXW7* (2%), meanwhile, 723 (94.5%) were predicted drivers carried by *NCOR1* (41%), *EP300* (28%), *TP53* (11%), *EZH2* (6%), *FBXW7* (6%), *HDAC3* (5%), and *PTEN* (3%) (Supplementary Table 43).

After identifying the oncogenic variants in the essential CR-related genes strongly involved in cancer, we then conducted an in silico drug prescription analysis. The aim of this analysis was to pinpoint suitable drugs targeting clinically actionable markers, using the Cancer Biomarker database^28^, which is an expanded version of a previous collection of genomic biomarkers related to anti-cancer drug response. Figure 6B depicts a Sankey plot of putative biomarkers involved in cancer treatments, indicating responsive effects. Patients with *EZH2* mutations (A692V, Y646C, Y646F, Y646H, Y646N, Y646S, and A682G) show responsiveness to tazemetostat. Oncogenic *FBXW7* mutations are receptive to steroids and mTOR inhibitors. The *PML-RARA* fusion responds to several treatments including tretinoin, a combination of tretinoin and arsenic trioxide, D-allose, and a combination of D-allose and arsenic. *PTEN* oncogenic mutations and *PTEN* deletions respond to everolimus. *PTEN* expression responds with sensitivity to trastuzumab, and a combination of gefitinib with erlotinib. Patients with *TOP2A* amplification respond to anthracyclines. Oncogenic *TP53* mutations, including R175H and R249, are receptive to alemtuzumab and doxorubicin. *TOP2A* expression is also receptive to doxorubicin. Lastly, patients with wildtype *TP53* respond to Unii-Q8MI0X869M, and a combination of oxaliplatin, capecitabine, and cetuximab (Supplementary Table 44).

### Drugs involved in late phase clinical trials

Figure 6C provides an update on the phase III and IV clinical trials where the essential CR-related proteins were involved according to the Open Targets Platform^29^. Of the 106 clinical trial events, 87% were in phase III and 13% in phase IV. Figure 6C also presents a Sankey plot that portrays 23 drugs with 8 mechanisms of action under investigation, targeting 10 essential CR-related proteins across 21 cancer types (Supplementary Table 45). Interestingly, only 10 (32%) of the essential CR-related proteins are currently being investigated in late phase clinical trials. This leaves 21 (68%) essential CR-related proteins that warrant further research to evaluate their potential for drug development.

## Discussion

The circadian clock, our near-24-h intrinsic biological timer, plays a pivotal role in coordinating time-dependent cellular processes and systemic physiology^1–3^. The growing knowledge of chronobiology has increased our understanding of how rhythm disruptions can trigger a range of health issues, from inadequate neurocognitive development, insomnia, and mental disorders, to cardiovascular diseases, metabolic dysfunctions, and cancer^5,15,16^. Many aspects of cancer biology, including its development and progression, can be understood through the chrono-disruption of essential CR-related proteins and signaling pathways. These disruptions can trigger uncontrolled proliferation, metastasis, inflammation, inhibition of apoptosis, immune evasion, angiogenesis, and resistance of anti-cancer drugs^3^. Within this context, the innovative strategy of drugging the clock—specifically targeting the circadian clock to treat cancer—opens promising avenues for cancer treatment. However, our understanding of the therapeutic targets and effective drugs involved in the circadian rhythms of cancer remains limited. To shed light on this, we conducted a comprehensive multi-omics data analysis prioritizing CR-related genes/proteins strongly associated with cancer pathogenesis and potential therapeutic strategies with responsive effects.

Our comprehensive and integrative analysis of CR-cancer scores across various multi-omics approaches enabled us to prioritize 31 essential CR-related genes/proteins with significant association to cancer. This assembled list provides a promising groundwork for potential therapeutic targets and highlights the profound role of the circadian rhythm in cancer pathogenesis. Among the prioritized entities, CSNK1D and CSNK1E emerged as key players, serving as canonical clock components directly involved in the generation and regulation of circadian rhythms and period length^30^. Their roles extend to various processes, including cell proliferation, DNA damage response, and cell migration^31^. Additionally, CDK1, TP53, PPARG, PTEN, ATG7, CREB1, PPP1CC, IL6, FBXL6, NAMPT, and PPP1CB constitute clock-controlled proteins. These proteins are downstream entities whose expression is driven by the rhythmic activity of the CCCs. The existence of such downstream control underscores the circadian clock’s influence over cellular processes and responses, highlighting the importance of timing in cellular regulation and the potential repercussions of its disruption. For instance, CDK1 plays a crucial role in cell division and cell cycle control. The regulation of CDK1 by the circadian clock influences cell cycle progression and cell proliferation rates^32^. TP53 regulates the expression of PER2 by blocking the ARNTL/CLOCK heterodimer and initiates cell cycle arrest or apoptosis in response to DNA damage^33^. Finally, we identified EZH2, HDAC2, HDAC3, GSK3B, PRKDC, FBXW7, PML, HDAC1, EP300, MAPK8, HNRNPU, DRD3, FBXL12, NCOR1, TOP2A, SIAH2, TARDBP, and NCOA2 as proteins involved in mediating neural mechanisms of circadian rhythmicity and its entrainment to environmental cues. These proteins aid in synchronizing our internal biological clocks with the external environment, underlining the intricate interplay between our bodies and our surroundings. For instance, PRKDC plays a significant role in DNA repair and influences the length of circadian rhythms^34^. FBXW7 regulates the degradation of NR1D1 and has associations with lipid/glucose homeostasis^35^. HNRNPU is crucial for expressing neuropeptides vital for SCN communication^36^, and its absence results in disrupted metabolic rhythms^37^. Collectively, these findings emphasize the importance of circadian rhythm regulation in various aspects of health and disease. They represent a substantial advancement in our understanding of how disruptions to the circadian rhythm can contribute to cancer development and progression.

Subsequently, our functional enrichment analysis highlighted the pivotal roles these 31 essential CR-related proteins play across various cancer-related biological processes and pathways. These proteins, for example, have a key role in programmed cell death, an essential process for maintaining cellular homeostasis. This process eliminates cells that may pose a threat due to DNA damage or uncontrolled proliferation^38^. Phosphorylation, a significant post-translational modification controlling protein function, was also identified. Any disruptions in this mechanism could result in abnormal protein function^39^. Moreover, these proteins play a significant role in cell differentiation and proliferation, both of which are fundamental for growth and development. Any disruption in these mechanisms could contribute to tumorigenesis^40^. Energy metabolism, another critical aspect of cellular function, was also highlighted. Given that alterations in energy metabolism are a recognized hallmark of cancer, understanding the role of these proteins could offer valuable insights into possible therapeutic strategies^41^. These essential CR-related proteins are also implicated in signaling pathways such as the apoptotic signaling pathway, a crucial self-destruction mechanism that cancer cells often evade^38^. Furthermore, their influence extends to the Notch, Wnt, and CKAP4 signaling pathways, crucial pathways involved in cancer development and progression due to their role in cell proliferation, differentiation, and survival^42–44^. These findings illustrate the diverse roles of these 31 essential CR-related proteins in numerous cancer-related biological processes and pathways. A comprehensive understanding of their functions and interactions will be critical in developing novel and effective strategies for cancer prevention, diagnosis, and treatment.

Interestingly, metastasis, another cancer hallmark phenotype, is strongly associated with disruptions in the circadian rhythm^45^. In our survival analysis of 25,775 patients with metastasis, we discovered that those with genomic alterations in 10 essential CR-related genes (*PTEN*, *TP53*, *EP300*, *NCOR1*, *GSK3B*, *FBXW7*, *PPARG*, *EZH2*, *CSNK1E*, and *TOP2A*) experienced a significantly shorter overall survival (31.64 months), compared to those without such alterations (55.72 months). Previous studies have also suggested a correlation between disruptions in circadian rhythm and patient survival^26^. This may be due to increased inflammation caused by cancer when rhythmic systems are disrupted^46–48^.

The prior identification of essential CR-related proteins and signaling pathways, which are closely associated with cancer pathogenesis, has enabled us to analyze the comprehensive landscape of pharmacogenomic clinical annotations and late-stage clinical trials to enhance cancer treatment efficacy. Regarding pharmacogenomic clinical annotations, the boostDM machine learning-based method^49^ identified 765 known and predicted oncogenic variants in 7 CR-related genes, also identified as clinically actionable targets for anti-cancer drugs. Furthermore, our in silico drug prescription analysis identified 21 pharmacogenomic clinical annotations with responsive effects, as shown in Fig. 6B. For instance, tazemetostat is recommended for patients with specific oncogenic mutations in *EZH2*. Steroids and mTOR inhibitors are beneficial for those with oncogenic mutations in *FBXW7*. For patients with a *PML*-*RARA* fusion, treatment options include tretinoin alone, tretinoin in combination with arsenic trioxide, D-allose alone, or D-allose with arsenic. Everolimus is suggested for those with *PTEN* oncogenic mutations and deletions. Patients with *PTEN* expression may be treated with trastuzumab or a combination of gefitinib and erlotinib. Anthracyclines are used for treating *TOP2A* amplification. Alemtuzumab is suitable for patients with *TP53* oncogenic mutations, whereas doxorubicin is recommended for those with specific *TP53* oncogenic mutations and *TOP2A* expression. Lastly, Unii-Q8MI0X869M or a combination of oxaliplatin, capecitabine, and cetuximab is beneficial for patients with *TP53* wildtype^28,50^.

In our review of clinical trials focusing on essential CR-related proteins, we found that 23 drugs are currently in phases III and IV. These drugs target 10 therapeutic entities and are being tested across 21 different cancer types. Our study revealed that topoisomerase inhibitors have shown a responsive effect on the TOP2A protein, while a GSK3 inhibitor has a similar effect on GSK3B. CDK1 responds to a CDK inhibitor, and CSNK1E to a PI3K delta inhibitor. Additionally, a methyltransferase inhibitor has been found effective on EZH2, and HDAC inhibitors have shown responses on HDAC1, HDAC2, and HDAC3. A PPAR receptor agonist has an impact on PPARG, while a dopamine receptor antagonist works on DRD3. However, out of the 31 essential CR-related proteins examined in our study, only 10 (32%) are currently targeted by drugs in these clinical trials. Thus, we propose that the remaining 21 CR-related proteins offer potential therapeutic targets for future cancer clinical trials.

In conclusion, our study elucidates an innovative therapeutic landscape oriented around the groundbreaking concept of drugging the clock. This approach, which targets the circadian clock for cancer treatment, underscores the vast potential of integrating circadian biology into oncology. This integration, enriched by pharmacogenomic approaches, patient-specific clinical data, and ethnicity considerations, not only enhances the precision, personalization, and effectiveness of cancer treatments^51–54^, but also mitigates side effects. Nonetheless, as this is an emerging field, further extensive research and rigorous clinical trials are imperative to validate the safety and efficacy of these interventions.

## Methods

### Gene/protein set

A set of 206 human genes/proteins associated with the “circadian rhythm” term was obtained from the Gene Ontology database (GO:0007623) (http://www.geneontology.org)^55,56^, and the David Bioinformatics Resource (https://david.ncifcrf.gov/)^57^. Subsequently, we manually curated and classified these CR-related genes/proteins in three categories: (a) CCCs, (b) CCGs/CCPs, and (c) genes/proteins involved in mediating the NMCRE. This classification was based on various gene ontology annotations. On the other hand, to determine which CR-related genes/proteins were already recognized as cancer drivers, we retrieved 874 driver genes/proteins from the Integrative OncoGenomics (intOGen) pipeline (https://www.intogen.org)^58^. Additionally, we utilized The Catalogue of Somatic Mutations in Cancer (COSMIC) Cancer Gene Census (CGC) (https://cancer.sanger.ac.uk/), which is an expert-curated repository of the genes/proteins known to drive cancer and is commonly employed in oncology research^59^.

### CR-cancer score

The CR-cancer score is a strategy for prioritizing the most relevant genes/proteins in each of the multi-omics approaches analyzed. This score involved assigning a rank from 1 to 0 to each gene/protein based on the ranking obtained from the results of each analysis. In the OncoPrint of the TCGA PanCancer Atlas, the CR-related genes were ranked based on the frequency of genomic and transcriptomic alterations. Genes with higher frequencies of alterations received a higher CR-cancer score. In the CR-PPi network, the CR-related proteins were ranked based on degree centrality. Proteins more connected to cancer driver proteins had higher degree centrality and therefore a higher CR-cancer score. In the unfavorable prognostic gene analysis, the genes with greater significance received a higher CR-cancer score. In the shortest pathways to cancer hallmark phenotype analysis, the CR-related proteins with lower distance scores to cancer phenotypes received a higher CR-cancer score. For the first time, the CR-cancer scores from each multi-omics approach were integrated to identify the most significantly associated essential CR-related genes/proteins with cancer pathogenesis. This prioritization strategy allowed the visualization of the top 10% (cutoff > 0.9) of genes/proteins in each of the multi-omics approaches.

### OncoPrint of genomic and transcriptomic alterations according to the TCGA PanCancer Atlas

After identifying the sets of CCCs, CCGs, and genes involved in mediating the NMCRE, we retrieved their genomic and transcriptomic alteration events in the PanCancer Atlas which belongs to TCGA consortium^60–62^. The genomic and transcriptomic alterations (mRNA upregulation, mRNA downregulation, CNV deep deletion, CNV amplification, fusion gene, inframe mutation, truncating mutation, and missense mutation) were analyzed in 10,918 individuals from 32 TCGA PanCancer types: GBM, UCS, UCEC, THCA, THYM, TGCT, SARC, PCPG, SKCM, PRAD, MESO, PAAD, OV, LUSC, LUAD, LIHC, KIRP, KICH, KIRC, HNSC, UVM, STAD, ESCA, CESC, LGG, BRCA, CRC, BLCA, CHOL, ACC, DLBC, and LAML. According to the Genomics Data Commons of the National Cancer Institute (https://portal.gdc.cancer.gov/) and the cBioPortal (http://www.cbioportal.org/)^63,64^, the mRNA upregulation and mRNA downregulation alterations were analyzed through RNA sequencing V2 RSEM where the expression Z-scores of tumor samples were compared to the expression distribution of all log-transformed mRNA expression of adjacent normal samples in each cohort^65^. The CNV amplifications and CNV deep deletions were identified using GISTIC2.0^66^; and the inframe, truncating, and missense driver mutations were identified through whole exome sequencing. Lastly, an “event” refers to the presence of at least one pathogenic alteration in a patient’s gene, and the sum of these events allows us to calculate the frequencies of genomic and transcriptomic alterations.

To design the OncoPrint, which encompasses the most significantly altered CR-related genes we: (a) calculated the number of alteration events per gene and per TCGA PanCancer type; (b) normalized the *f* of alteration events dividing the number of alterations per gene by the number of individuals per each cancer cohort; (c) calculated the mean *f* per gene and per alteration type considering all PanCancer types; (d) identified the most altered genes taking into account as a cutoff the mean *f* of all genes; and (e) validated the most significant altered genes comparing the alteration frequency events between the group of genes with the highest alteration frequencies (cutoff > mean *f*) versus the group of genes with the lowest alteration frequencies (cutoff < mean *f*) by using the Mann–Whitney U test (*P* < 0.001). We also applied the Bonferroni correction test (*P* < 0.001) to perform a multiple comparison between (g) the whole TCGA PanCancer alterations, and (h) the TCGA PanCancer Atlas^62,67–70^. Lastly, we calculated the CR-cancer score to prioritize the top 10% (cutoff > 0.9) of genes with the most genomic and transcriptomic alterations across the TCGA PanCancer Atlas.

### Circadian rhythm protein–protein interactome network

The CR-PPi was performed to enhance our understanding of the connectivity between CCCs, CCPs, proteins involved in mediating the NMCRE, and cancer driver proteins. This analysis utilized the human proteome data from the Cytoscape StringApp, considering only the highest confidence interactions (cutoff > 0.9) based on experimental evidence^71–73^. To determine the degree centrality, which indicates the number of edges connected to each node in a network^74–77^, we employed the CytoNCA app^78^. The nodes and edges were then organized using the organic layout, and the CR-PPi network was visualized through the Cytoscape software v.3.10^71,72^. The analysis included all CR-related proteins with at least one high-confidence interaction in the human proteome. For the inclusion of cancer driver proteins in the CR-PPi network, we sourced the data from the intOGen pipeline and the COSMIC-CGC database^58,59^. Lastly, we compared the average degree centrality among CCCs, CCPs, proteins involved in mediating the NMCRE, and cancer driver proteins; and we calculated the CR-cancer score to prioritize the top 10% (cutoff > 0.9) of proteins with the highest degree centrality in the CR-PPi network.

### A pathology atlas of the human cancer transcriptome

The Human Pathology Atlas, created as part of the Human Protein Atlas program (https://www.proteinatlas.org/humanproteome/pathology), has been used to explore the prognostic role of each CR protein-coding gene across 17 TCGA PanCancer types in nearly 8000 patients. The Human Pathology Atlas uses transcriptomics and antibody-based profiling to provide a standalone resource for precision medicine in cancer^23^. Immunohistochemistry (IHC) is the gold standard methodology for in situ protein expression analysis in tissue samples. The combination of IHC and tissue microarray (TMA) technology allows simultaneous analysis of hundreds of tissue samples with an unprecedented degree of experimental standardization^79^.

Staining profiles for proteins in human tumor tissue based on immunohistochemistry using TMA and log-rank *P*-value for Kaplan–Meier analysis of correlation between mRNA expression level and patient survival were produced. Patient samples were classified into two expression groups and the correlation between expression level and patient survival was examined. The prognosis of each group of patients was examined by Kaplan–Meier survival estimators, and the survival outcomes of the two groups were compared by log-rank tests. CCCs, CCGs, and genes involved in NMCRE with log rank *P*-values less than 0.001 in maximally separated Kaplan–Meier analysis were defined as unfavorable prognostic genes^80^. Lastly, we calculated the CR-cancer score to prioritize the top 10% (cutoff > 0.9) of the most significant unfavorable prognostic genes.

### Shortest pathways from circadian rhythm proteins to cancer hallmark phenotypes

CancerGeneNet, available at (https://signor.uniroma2.it/CancerGeneNet/), is a curated bioinformatics resource provided by SIGNOR^81^. This resource utilizes experimental annotations to infer potential causal pathways linking proteins to various cancer hallmark phenotypes^24^. Iannuccelli et al., programmatically implemented the calculation of shortest distance scores or paths from specific proteins to cancer phenotypes using the shortest path function from the *igraph* R package. Thus, our objective was to investigate the signaling crossroad between CR-related proteins and cancer^45^.

In this context, we computed the shortest paths for positive or negative regulations of CR-related proteins associated with angiogenesis, immortality, inflammation, metastasis, proliferation, cell death, differentiation, DNA repair, and glycolysis. Subsequently, we conducted a multiple comparison test using the Bonferroni correction (*P* < 0.001, 95% confidence interval) to compare the distance scores of CR-related proteins across different cancer phenotypes. Finally, we calculated the CR-cancer score to prioritize the top 10% (cutoff > 0.9) of the CCCs, CCPs, and proteins mediating NMCRE with the shortest paths to each cancer hallmark phenotype.

### Multi-omics data integration and functional enrichment analysis

The functional enrichment analysis gives scientists curated signatures and interpretation of protein sets from omics-scale experiments^25,82^. Therefore, we performed the functional enrichment analysis to the essential CR-related proteins most significantly associated with cancer identified through the integration of CR-cancer scores (cutoff > 0.9) per multi-omics approach. The enrichment was analyzed using g:Profiler version e101_eg48_p14_baf17f0 (https://biit.cs.ut.ee/gprofiler/gost)^25^ to obtain significant annotations (Benjamini–Hochberg FDR* q* < 0.001) related to GO biological processes (http://geneontology.org/)^55^, the Reactome signaling pathways (https://reactome.org/)^83^, and WikiPathways (https://www.wikipathways.org/)^84^. The functional enrichment analysis was visualized through a Manhattan plot, and significant terms related to cancer hallmark phenotypes were manually curated.

### Pharmacogenomic clinical annotations of circadian rhythm genes in cancer

We set out to further evaluate the panoramic landscape of cancer pharmacogenomic strategies where the essential CR-related genes were involved. Accordingly, we identified known and predicted oncogenic variants highly associated with cancer. Following this, we performed an in silico drug prescription analysis to reveal several clinically actionable genes directly targeted by anti-cancer drugs^27^.

The identification of oncogenic variants was a two-step process. Initially, we extracted 27,893 single nucleotide variants and insertion/deletion variants belonging to the CR-related genes that are significantly involved in cancer. These were retrieved from the Genome Aggregation database (gnomAD v3.1.2) (https://gnomad.broadinstitute.org/), using GRCh38 as the human genome reference^85,86^. Then, we performed boostDM, a machine learning-based methodology integrated into the Cancer Genome Interpreter (CGI) platform (https://www.cancergenomeinterpreter.org/home), to conduct an in silico saturation mutagenesis of cancer genes. This process allowed us to assess the oncogenic potential of the previously mentioned mutations in human tissues^49,50^. Lastly, boostDM categorizes mutations as known drivers or predicted drivers.

Furthermore, the in silico drug prescription integrated into the CGI includes putative biomarkers of drug responses found in the tumor, organized according to various levels of clinical relevance. The CGI utilizes two key resources to define the association between oncogenic variants and drug responses: the Cancer Biomarker database and the Cancer Bioactivities database^28,50^. Finally, we carried out an in silico analysis to determine the druggability of driver variants located within CR-related genes. This allowed us to identify the most effective precision oncology treatments^87^.

### Drugs involved in late phase clinical trials

Regarding the identification of potential therapeutic targets and drugs in late phase clinical trials for cancer, we analyzed data from The Open Targets Platform (https://www.targetvalidation.org). This platform provides comprehensive data integration, allowing for access to and visualization of potential drug targets associated with cancer. It showcases all drugs in clinical trials linked to the essential CR-related proteins significantly involved in cancer^29,88^. Furthermore, the Broad Institute’s Drug Repurposing Hub (https://clue.io/repurposing) is a curated collection of drugs approved by the Food and Drug Administration (FDA), along with drugs in clinical trials and preclinical tool compounds^89^. This bioinformatics tool facilitated our identification of the mechanisms of action of drugs used in cancer treatment^89^.

### Supplementary Information

Supplementary Tables.

## Data Availability

All data generated or analyzed during this study are included in this published article (and its Supplementary Information files).
